# Effect of Psychological–Behavioral Intervention on the Depression and Anxiety of COVID-19 Patients

**DOI:** 10.3389/fpsyt.2020.586355

**Published:** 2020-11-20

**Authors:** Xiangyu Kong, Fanyang Kong, Kailian Zheng, Min Tang, Yi Chen, Jiahuan Zhou, Yi Li, Le Diao, Shouxin Wu, Piqi Jiao, Tong Su, Yuchao Dong

**Affiliations:** ^1^Huoshenshan Hospital, Wuhan, China; ^2^Department of Gastroenterology, Shanghai Changhai Hospital, Shanghai, China; ^3^Shanghai Zhangjiang Institute of Medical Innovation, Shanghai Biotecan Pharmaceuticals Co., Ltd., Shanghai, China; ^4^College of Psychology, Naval Medical University, Shanghai, China

**Keywords:** COVID-19, depression, anxiety, social support, psychological-behavioral intervention

## Abstract

The COVID-19 epidemic has caused increasing public panic and mental health stress. In this study, we explore the prevalence and factors linked to anxiety and depression in hospitalized patients with COVID-19. A total of 144 patients diagnosed with COVID-19 underwent depression and anxiety assessment by using the Hospital Anxiety and Depression Scale (HADS). Social support level was also evaluated by the Perceived Social Support Scale (PSSS) at admission. Results showed that gender, age, oxygen saturation, and social support were associated with anxiety for COVID-19 patients. In addition, age, family infection with SARS-CoV-2, and social support were the risk factors associated with depression. Moreover, we designed a psychological–behavioral intervention (PBI) program that included psychological support and breathing exercises, and explored its effects on patients with COVID-19. Of the 144 participants, 26 patients with both anxiety and depression symptoms (cutoff score of ≥8 on HADS-A and HADS-D) were randomly assigned to the intervention group and the control group at a 1:1 ratio. After 10-day treatment, the HADS scores of depression and anxiety were significantly reduced in the intervention group, and PSSS scores were also significantly improved. However, no significant differences in HADS and PSSS scores between pre- and post-treatment were found in the control group. Our findings indicate that mental concern and appropriate intervention are essential parts of clinical care for COVID-19 patients.

## Introduction

Since December, 2019, an outbreak of coronavirus disease 2019 (COVID-19), caused by the severe acute respiratory syndrome coronavirus 2 (SARS-CoV-2), has widely and rapidly spread in China and around the world (1). As of June 21, 2020, more than 8,700,000 confirmed cases and at least 460,000 deaths have been reported in 216 countries (territories/areas), according to the World Health Organization (WHO) (2). The grim epidemic has caused increasing public panic and mental health stress. Mental health is becoming an issue that cannot be ignored, while trying to control the outbreak.

Previous studies have shown that depression and anxiety are common and persistent mental illness in various illnesses including chronic diseases (3, 4) and cancer (5). These studies indicated that patients with mental illness, including depression and anxiety, may have difficulty with symptom control, as well as impaired quality of life. However, recently published researches on psychological impact of COVID-19 are mainly focused on healthcare workers (6, 7) and the general public (8), who were worried about the risks of infection and protective measures. Note that patients after diagnosis of COVID-19 were more likely to have psychological concerns such as fear of progression of their illness, disability, or premature death. Additionally, it has been reported that psychological distress may affect patient compliance with medical treatment (9, 10) and disease duration (4, 11). Therefore, it is vital to pay attention to the mental health of COVID-19 patients, and appropriate intervention may be beneficial for them. However, so far, the prevalence and related factors of anxiety and depression in patients infected with COVID-19 has been rarely reported.

It has been demonstrated that a psychological intervention can reduce emotional distress, promote positive health habits, and enhance immune responses for patients with cancer and other diseases (12–14). As for infectious diseases, optimism and related constructs could improve the anxiety control and life quality of chronic hepatic B patients (15), as well as the pain management in people with HIV (16). We thought that psychological intervention may be beneficial for patients' mental health and therapeutic process. Given that the doctors involved in the fight against the COVID-19 were not professional psychologists, we mainly referred to U.S. SPIKES (17) and Australian Consensus Guidelines (18) on the strategies for dealing with patients' negative emotions, making the intervention protocol operable for clinical staff. Meanwhile, breathing exercises have been reported to reduce the levels of anxiety and depression and improve pulmonary function (19, 20). Hence, we designed a psychological–behavioral intervention (PBI) program that included psychological support and breathing exercises for patients with anxiety and depression. We intend to investigate whether this kind of intervention could effectively lower anxiety and depression level of patients.

From the above, two aims were included in the present study. Aim 1: To explore the prevalence and factors linked to anxiety and depression in hospitalized patients with COVID-19. Aim 2: To determine the effect of PBI on anxiety and depression of patients with COVID-19.

This study may draw more attention to the psychological state of patients with COVID-19 and assist doctors to provide more appropriate treatment and psychological interventions to improve mental and physical health of patients during the campaign to contain and eradicate COVID-19.

## Methods

### Prevalence and Factors Linked to Anxiety and Depression in Hospitalized Patients With COVID-19

#### Participants

Patients were admitted to two divisions (Division 1 of the Second Department and Division 2 of the Fourth Department) of Huoshenshan Hospital (Wuhan, China) from 23 February 2020 to 13 March 2020. The inclusion criteria were as follows: (1) aged 15–85 years; (2) patients were diagnosed with COVID-19 according to WHO interim guidance. The exclusion criteria were as follows: (1) patients with intellectual and cognitive impairment; (2) patients did not have a smartphone. Informed consent was provided by subjects before study commencement.

**Figure 1:**
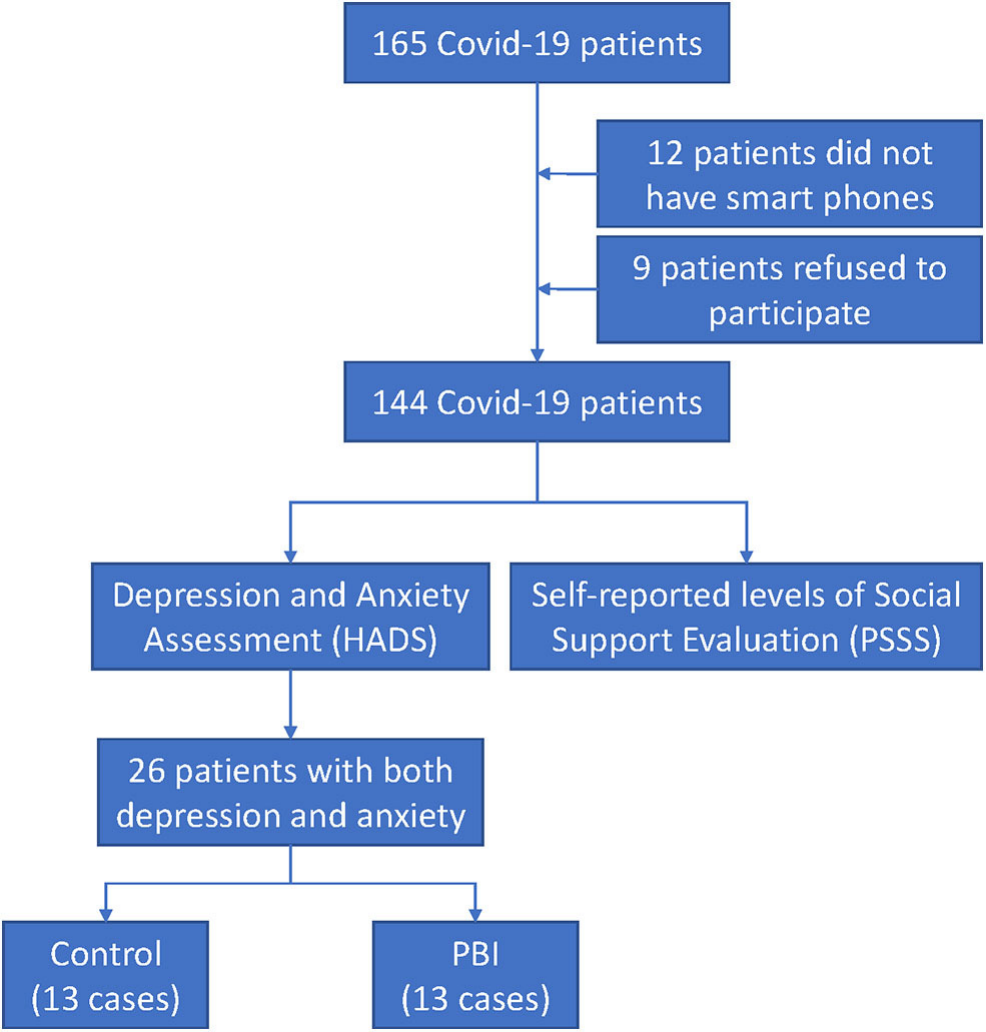
A flow diagram indicating study design.

The flow diagram (Figure 1) shows that a total of 165 patients were admitted to two divisions of Huoshenshan Hospital during that period. Nine patients refused to participate in the research study, and 12 patients were subsequently excluded due to not having smartphones. Eventually, 144 patients with confirmed COVID-19 completed the questionnaires through an online survey platform (“SurveyStar,” Changsha Ranxing Science and Technology, Shanghai, China) at admission. The study was approved by the Research Ethics Commission of Huoshenshan Hospital.

#### Assessments

##### Hospital Anxiety and Depression Scale (HADS)

The HADS is a self-assessment questionnaire designed by Zigmond et al. in 1983, which aims to detect anxiety and depression symptoms in general hospital patients. It has been acknowledged that the Chinese version (published in 1993) of the HADS had good internal consistency and favorable scale equivalence (21). The degree of anxiety and depression is rated by the accumulated scores: score 0–7, indicating no anxiety or depression; score 8–10, indicating mild levels of anxiety or depression; score 11–14, indicating moderate levels of anxiety or depression; and score 15–21, indicating severe levels of anxiety or depression.

##### Perceived Social Support Scale

The 12-item PSSS was compiled by Zimet et al. in 1987. The Chinese version PSSS (published in 1996) has been widely adopted to measures with perceived support from family, friends, and other ways in the Chinese population (22). Total scores range from 0 to 84, classified into low (12–36), moderate (37–60), and high levels of social support (61–84).

### Effect of PBI on Patients With COVID-19

#### Study Design

This study is a single-center, evaluator-blinded, randomized controlled trial.

#### Participants

Because all 144 participants completed the HADS questionnaires through an online survey platform “SurveyStar” at admission. We could obtain the scores of each patient once they finished the test. We consecutively recruited the patients with both symptoms of anxiety and depression in the PBI study. A cutoff score of ≥8 on both anxiety and depression subscales was applied to identify patients with both anxiety and depression.

Of the 144 participants, twenty-six patients with COVID-19 were identified with both symptoms of anxiety and depression via HADS questionnaire.

#### Randomization

Twenty-six eligible patients were consecutively and randomly assigned to the PBI group and the control group (13 patients in each group), according to the order of admission. All of them signed the informed consents. There was no difference in the age and sex distribution between the control group and the intervention group. Each patient was isolated in a separate room at the Huoshenshan Hospital. The intervention group and the control group have no chance to communicate with each other about the treatment.

#### Intervention

All patients were given their normal medical regimens and basic care during hospitalization. For the control group, they communicated with the doctors only on daily ward rounds. While for the intervention group, a 10-day PBI program was carried out when stable status of patients was confirmed after admission.

The details of PBI were as follows:

1. Breathing exercise:

Every morning, two trained medical workers would guide patients to have a breathing exercise for 20 min around 10:00 a. m. (Supplementary Figure 1). The breathing exercise is based on Yoga's breathing techniques and focuses on stimulating nasal and diaphragmatic breathing, increasing the expiratory time, slowing the respiratory flow, and regulating the breathing rhythm.

2. Psychosocial support:

In the present study, we mainly referred to U.S. SPIKES (17) and Australian Consensus Guidelines (18) on the psychosocial support protocols for delivering bad news to patients. We consulted with psychologists to develop the procedure for psychological intervention. Meanwhile, five psychological experts were invited to provide scientific suggestions and feasibility assessment for the psychological intervention.

The psychological intervention process includes:

1. Setting up interview
2. Encouraging patients to express feelings
3. Expressing understanding and comfort patients
4. Giving knowledge and information about COVID-19
5. Providing some simple relaxation techniques, and offering the self-emotional management skills (such as listening to music as a way of distraction when in a bad mood)
6. Summary (helping patients to eliminate mental tension and build up confidence to overcome disease, as well as persuading them to cooperate with treatment and care in a positive and optimistic manner)

The psychological support intervention was designed to be brief within 15 min, considering the limited condition (medical workers needed to wear masks and protective clothing in isolation wards) in communication with patients.

The psychological intervention was performed by two appointed medical staffs, who have been trained for providing the psychological support.

1. Regular training: Before being temporarily assigned to Huoshenshan Hospital, the two appointed medical staffs were medical workers of Changhai Hospital affiliated to Navy Medical University. They have received regular doctor–patient communication training, including lectures on “Common psychological problems with patients” and “How to better communicate with patients” by psychologists at the Naval Medical University.
2. Guidance by Psychological Intervention Manual: After the outbreak of COVID-19, professors from the College of Psychology of the Naval Medical University compiled the “COVID-19 Psychological Guidance Manual.” This manual introduced potential psychological response of patients during the epidemic, and some techniques of psychological care. The two appointed medical staffs studied the manual and held telephone sessions with psychologists, who gave more details about psychological support skills.

The procedure of the psychological intervention was jointly designed by researchers (including the two appointed medical staffs) and psychological experts, according to actual situation of Huoshenshan Hospital. When problems appeared in the implementation process, remote assistance would be given by psychological experts via video calls.

#### Assessments

##### Hospital Anxiety and Depression Scale (HADS)

After a 10-day treatment, anxiety and depression of patients were assessed again by use of HADS. The HADS-A (Hospital Anxiety and Depression Scale-Anxiety) score and HADS-D (Hospital Anxiety and Depression Scale-Depression) scores were used as indexes to evaluate the intervention effects.

##### Perceived Social Support Scale

After a 10-day treatment, self-reported levels of social support were assessed again by use of PSSS.

### Statistical Analysis

SPSS software, version 19 were used for statistical analysis. Means and proportions of the given data for each variable were calculated. Categorical variables were analyzed using the Pearson's chi-square test or Fisher's exact test. Continuous variables were analyzed using non-paired Student *t*-test or paired Student *t* test. Multivariate regression analysis with stepwise method was performed to identify factors associated with depression and anxiety. Multivariate analysis of variance was used to analyze the difference between the PBI group and the control group in the post-treatment HADS score. Differences between groups were considered to be significant when the *p*-value was < 0.05.

## Results

### Prevalence and Factors Linked to Anxiety and Depression in Hospitalized Patients With COVID-19

#### Demographic Characteristics

A total of 165 patients were admitted to two divisions of Huoshenshan Hospital (Wuhan, China) from 23 February 2020 to 13 March 2020. The flow diagram (Figure 1) shows that nine patients refused to participate in the research study and 12 patients were subsequently excluded due to not having smartphones. A total of 144 participants, including 70 male and 74 female, were eligible and completed the questionnaires in the current study. The age of the study participants ranged from 15 to 87 years. Their average age was 49.98 ± 13.73 years. Participants were mostly living with a spouse (121/144, 84%). About a third of the subjects (54/144, 37.5%) were well educated (≥bachelor's degree), and only 4 of 144 participants (2.8%) had primary education. Oxygen saturation is a key clinical index for evaluating the severity of patients with COVID-19 (23). In the present study, 11.1% of participants who had an oxygen saturation ≤93% at rest were with severe disease. Other clinical symptoms of COVID-19 patients were also recorded. As other COVID-19-related studies reported (1, 24–26), fever (84%), cough (78.5%), and shortness of breath (50.7%) were the most common symptoms. In addition, considering that other family members' infection may cause emotional distress to the participants, we also collected the infection status of family members. Fifty-nine participants (41%) had one or more family members infected. Demographic characteristics are listed in Table 1.

**Table 1:** Baseline demographic and clinical characteristic of patients with COVID-19.

	***n***	**%**
**Gender**
Male	70	48.6
Female	74	51.4
**Age (years)**
≤ 50	70	48.6
>50	74	51.4
**Marital status**
Married	121	84.0
Single	17	11.8
Divorced	2	1.4
Widowed	4	2.8
**Education status**
Primary	4	2.8
Lower secondary	34	23.6
Upper secondary	52	36.1
University/master/doctorate	54	37.5
**Oxygen saturation at rest**
≤ 93%	16	11.1
>93%	128	88.9
**Infection status of family members**
Infected	59	41.0
Non-infected	85	59.0
**Clinical symptoms**
Fever	121	84.0
Cough	113	78.5
Shortness of breath	73	50.7
Fatigue	63	43.8
Chest distress	28	19.4
Myalgia	14	9.7

#### Psychosocial Characteristics of the Participants With COVID-19

The mean score of anxiety subscale and depression subscale for all patients was 6.35 ± 4.29 and 5.44 ± 4.32, respectively. With the reference to HADS, 50 (34.72%) and 31 (28.47%) participants presented symptoms of anxiety and depression, respectively. Regarding the patients' anxiety levels, it was found that 17.36, 12.5, and 4.86% appeared to have mild, moderate, and severe anxiety, respectively. As for the depression levels of patients, 20 were mildly depressed (13.89%), 15 were moderately depressed (10.42%), and 6 were severely depressed (4.17%).

#### Correlations Among Depression, Anxiety, and Social Support in COVID-19 Patients

There is a large body of evidence that shows that social support plays a beneficial role in mental health (27). Self-reported levels of social support were assessed among the patients with COVID-19. The mean social support score for all participants was 63.41 ± 11.99. The average score of family, friends, and other support was 22.35 ± 4.42, 20.53 ± 4.60, and 20.52 ± 4.55, respectively. More than half of the participants (90/144, 62.5%) exhibited high level of perceived social support.

The bivariate correlations showed that less social support was correlated with more anxious (*r* = −0.196, *p* < 0.05) and depressive (*r* = −0.360, *p* < 0.05) symptoms (Table 2). In detail, friend support (*r* = −0.165, *p* < 0.05) and other support (*r* = −0.230, *p* < 0.05) were significantly negatively correlated with anxiety. In addition, family support (*r* = −0.283, *p* < 0.05), friend support (*r* = −0.307, *p* < 0.05), and other support (*r* = −0.363, *p* < 0.05) were significantly negatively correlated with depression.

**Table 2:** Association between anxiety, depression, and social support.

**Anxiety**	**Depression**	**Social support**	**Family support**	**Friend support**	**Other supports**
Anxiety	0.512^**^	−0.196^*^	−0.124	−0.165^*^	−0.230^**^
Depression		−0.360^**^	−0.283^**^	−0.307^**^	−0.363^**^
Social support			0.881^**^	0.875^**^	0.896^**^
Family support				0.642^**^	0.702^**^
Friend support					0.671^**^
Other supports					

* *p < 0.05*,

** *p < 0.01*.

#### Factors Associated With Depression and Anxiety Among Patients With COVID-19

In order to investigate the factors related to depression and anxiety among patients with COVID-19, anxiety and depression scores were compared between different groups. As shown in Table 3, anxiety and depression scores were significantly higher in those who were older (age > 50) and with low education. Additionally, patients with lower oxygen saturation had higher anxiety score, and those getting less social support had higher depression scores.

**Table 3:** Comparison of anxiety and depression scores on different variables (*N* = 144).

	**Anxiety score**					**Depression score**				
	**Mean ± SD**	***t***	**df**	***p***	**Mean difference (95% CI)**	**Mean ± SD**	***t***	**df**	***p***	**Mean difference (95% CI)**
**Gender**										
Male	5.71 ± 3.98	−1.752	142	0.082	−1.245 (−2.650 to 0.160)	5.47 ± 4.30	0.073	142	0.942	0.053 (−1.379 to 1.484)
Female	6.96 ± 4.51					5.42 ± 4.39				
**Age (years)**										
≤ 50	4.91 ± 3.40	−4.129	142	** < 0.001**	−2.802 (−4.143 to −1.460)	4.33 ± 4.44	−3.098	142	**0.002**	−2.171 (−3.557 to −0.786)
>50	7.72 ± 4.62					6.50 ± 3.97				
**Marital status**										
Married	6.53 ± 4.24	1.122	142	0.264	1.094 (−0.834 to 3.023)	5.50 ± 4.13	0.326	142	0.745	0.322 (−1.631 to 2.275)
Single/divorced/widowed	5.43 ± 4.54					5.17 ± 5.36				
**Education level**										
Primary/secondary	7.09 ± 4.66	2.710	142	**0.008**	1.959 (0.530 to 3.389)	6.06 ± 4.47	2.217	142	**0.028**	1.630 (0.176 to 3.083)
University/master/doctorate	5.13 ± 3.30					4.43 ± 3.92				
**Oxygen saturation at rest**										
≤ 93%	8.75 ± 5.88	2.407	142	**0.017**	2.695 (0.482 to 4.909)	6.50 ± 5.53	1.035	142	0.303	1.188 (−1.081 to 3.456)
>93%	6.05 ± 3.98					5.31 ± 4.16				
**Infection status of family members**										
Infected	6.92 ± 4.33	1.310	142	0.192	0.951 (−0.484 to 2.385)	6.19 ± 4.63	1.726	142	0.087	1.257 (−0.183 to 2.697)
Non-infected	5.96 ± 4.25					4.93 ± 4.05				
**Social support**										
High	5.89 ± 4.28	1.690	142	0.093	−1.241 (−2.692 to 0.211)	4.53 ± 3.89	3.377	142	**0.001**	−2.430 (−3.852 to −1.008)
Low–Moderate	7.13 ± 4.23					6.96 ± 4.63				

*95% CI, 95% confidence interval. The values in bold mean statistically significant*.

The multiple linear regression analysis (Table 4) showed that gender (β = 1.446, *p* = 0.034), age (β = 0.074, *p* = 0.003), oxygen saturation (β = −2.140, *p* = 0.049), and social support (β = −1.545, *p* = 0.017) were associated with anxiety for COVID-19 patients. It suggested that female, and patients who are older, with lower oxygen saturation, and less social support would tend to present anxiety symptoms. Moreover, age (β = 0.084, *p* = 0.001), family infection with SARS-CoV-2 (β =1.515, *p* = 0.027), and social support (β = −2.236, *p* < 0.001) were the factors associated with depression. The results indicate that patients with older age, family member infection, and less social support are more likely to be depressive (Table 4).

**Table 4:** Multivariate regression analysis of factors associated with anxiety and depression.

	**Unstandardized coefficients**			**Standardized coefficients**	***t***	***p***
	***b***	**95% CI for *b***	**SE**	**β**		
**Anxiety**^a^						
Gender (male/female)	1.446	0.111 to 2.780	0.675	0.169	2.142	**0.034**
Age	0.074	0.025 to 0.123	0.025	0.236	2.987	**0.003**
Oxygen saturation (≤ 93%/>93%)	−2.140	−4.268 to −0.012	1.076	−0.157	−1.988	**0.049**
Social support (low/moderate/high)	−1.545	−2.804 to −0.286	0.637	−0.191	−2.427	**0.017**
**Excluded variables**						
Marital status (married/other)	0.029	–	–	–	0.327	0.744
Infection status of family members (yes/no)	−0.128	–	–	–	−1.618	0.108
Education level	0.045	–	–	–	0.547	0.585
**Depression**^b^						
Age	0.084	0.035 to 0.132	0.024	0.266	3.429	**0.001**
Infection status of family members (yes/no)	1.515	0.172 to 2.858	0.679	0.173	2.230	**0.027**
Social support (low/moderate/high)	−2.236	−3.477 to −0.996	0.627	−0.275	−3.564	** <0.001**
**Excluded variables**						
Gender (male/female)	0.004	–	–	–	0.052	0.959
Marital status (married/other)	0.141	–	–	–	1.617	0.108
Oxygen saturation (≤ 93%/>93%)	−0.051	–	–	–	−0.646	0.519
Education level	−0.003		–	–	−0.040	0.968

*95% CI: 95% confidence interval*.

a *Dependent variable: anxiety score*.

*Predictive variables tested by multiple linear regression (stepwise method): Gender, Age, Oxygen saturation, and Social support*.

*R^2^ = 0.153, F = 6.274, p = 0.000*.

b *Dependent variable: depression score*.

*Predictive variables tested by multiple linear regression (stepwise method): Age, Infection status of family members, and Social support*.

*R^2^ = 0.169, F = 9.469, p = 0.000*.

*The values in bold mean statistically significant*.

### The Effect of PBI on Patients With COVID-19

#### The Effect of PBI on Anxiety and Depression of Patients With COVID-19

Of the 144 participants, 26 patients with COVID-19 were identified with both symptoms of anxiety and depression via HADS questionnaire. They were consecutively and randomly assigned to the PBI group and the control group according to the order of admission. Figure 1 shows that there were 13 patients in each group. There was no significant difference in baseline scores of anxiety and depression between the control group and the PBI group (*p* = 0.244 and *p* = 0.431, respectively) (Table 5). The mean score of anxiety and depression for the control group was 11.23 ± 3.219 and 10.77 ± 2.948. For the PBI group, the mean score of anxiety and depression was 12.62 ± 2.663 and 11.69 ± 2.926, respectively.

**Table 5:** Comparison of anxiety and depression level between the PBI group and the control group.

	**Number of patients**					**Score**					
	**PBI (*N* = 13)**	**Control (*N* = 13)**	**Chi-square value**	**df**	***p*^a^**	**PBI (*N* = 13)**	**Control (*N* = 13)**	**Mean difference (95%CI)**	***t***	**df**	***p*^b^**
**Pre-treatment**											
Anxiety	13	13	–	–	–	12.62 ± 2.663	11.23 ± 3.219	1.385 (−1.006 to 3.776)	1.195	24	0.244
Depression	13	13	–	–	–	11.69 ± 2.926	10.77 ± 2.948	0.923 (−1.455 to 3.301)	0.801	24	0.431
**Post-treatment**											0.006^c^
Anxiety	3	8	3.939	1	0.111	6.15 ± 3.579	9.92 ± 3.707	−3.769 (−6.719 to −0.820)	−2.637	24	0.014
Depression	3	9	5.571	1	0.047	5.92 ± 3.730	9.38 ± 2.785	−3.462 (−6.126 to −0.797)	−2.681	24	0.013

a *Pearson chi-square test or Fisher's exact test*.

b *Student t-test for independent samples*.

c *Multivariate analysis of variance (MANOVA), F = 6.539, Hypothesis df = 2, Error df = 23, p = 0.006*.

*PBI, psychological–behavioral intervention*.

**Figure 2:**
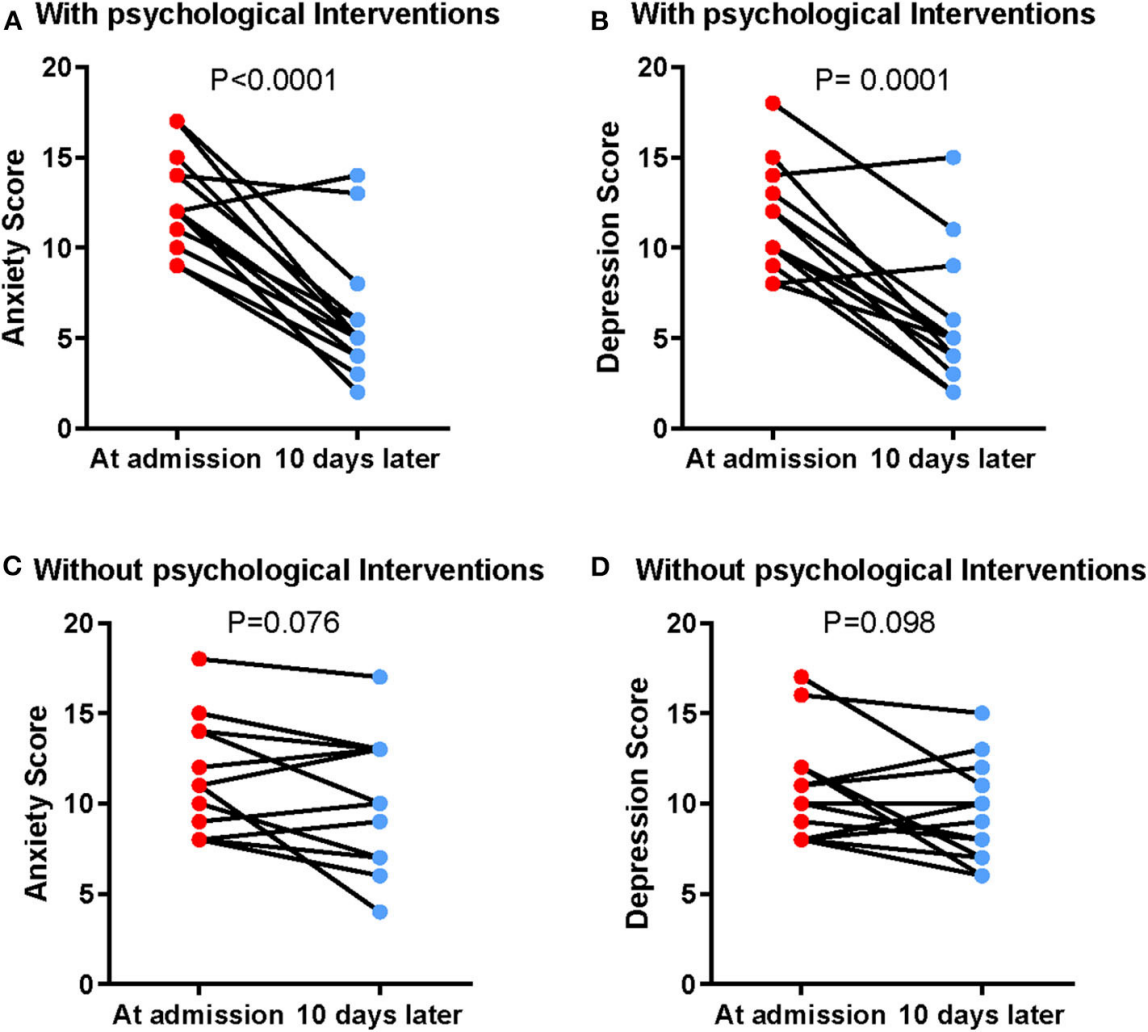
Reduced anxiety and depression by PBI in patients with COVID-19. The alteration of HADS-A score in the intervention group **(A)** and control group **(B)**. The alteration of HADS-D score in the intervention group **(C)** and control group **(D)**.

After a 10-day PBI treatment, the HADS-A score (6.15 ± 3.579) and HADS-D score (5.92 ± 3.730) were significantly reduced in the intervention group (*p* < 0.0001 and *p* = 0.0001, respectively) (Figures 2A,B and Table 6), whereas the HADS-A score (9.92 ± 3.707) and HADS-D score (9.92 ± 3.707) of the control group were not significantly different after 10-day hospitalization (*p* = 0.076 and *p* = 0.098, respectively) (Figures 2C,D and Table 6). Additionally, the multivariate analysis of variance showed that there was significant difference between the PBI group and the control group in the post-treatment HADS score (*p* = 0.006, Table 5). HADS-A score and HADS-D score were significantly lower in the PBI group than those in the control group after 10-day treatment (*p* = 0.014 and *p* = 0.013, respectively) (Table 5).

**Table 6:** Comparison of anxiety and depression scores between pre- and post-treatment in the PBI group and the control group.

		**Pre-treatment**	**Post-treatment**	**Mean difference (95% CI)**	***t***	**df**	***p*^a^**
PBI	HADS-A	12.62 ± 2.663	6.15 ± 3.579	6.462 (4.152 to 8.771)	6.097	12	<0.0001
	HADS-D	11.69 ± 2.926	5.92 ± 3.730	5.769 (3.631 to 7.908)	5.877	12	0.0001
Control	HADS-A	11.23 ± 3.219	9.92 ± 3.707	1.308 (−0.160 to 2.775)	1.942	12	0.076
	HADS-D	10.77 ± 2.948	9.38 ± 2.785	1.385 (−0.298 to 3.068)	1.793	12	0.098

a *Paired Student t-test*.

*HADS-A, Hospital Anxiety and Depression Scale-Anxiety score; HADS-D, Hospital Anxiety and Depression Scale-Depression score*.

The number of anxious patients after intervention was three, which was lower (*p* = 0.111) compared with that in the control group (*n* = 8) (Table 5). Additionally, there were three depressed patients in the intervention group after PBI, which was less compared with that in the control group (*n* = 9) (*p* = 0.047) (Table 5). The above data indicate that PBI is effective in reducing anxiety and depression level in patients with COVID-19.

#### The Effect of PBI on Social Support Level of Patients With COVID-19

**Figure 3:**
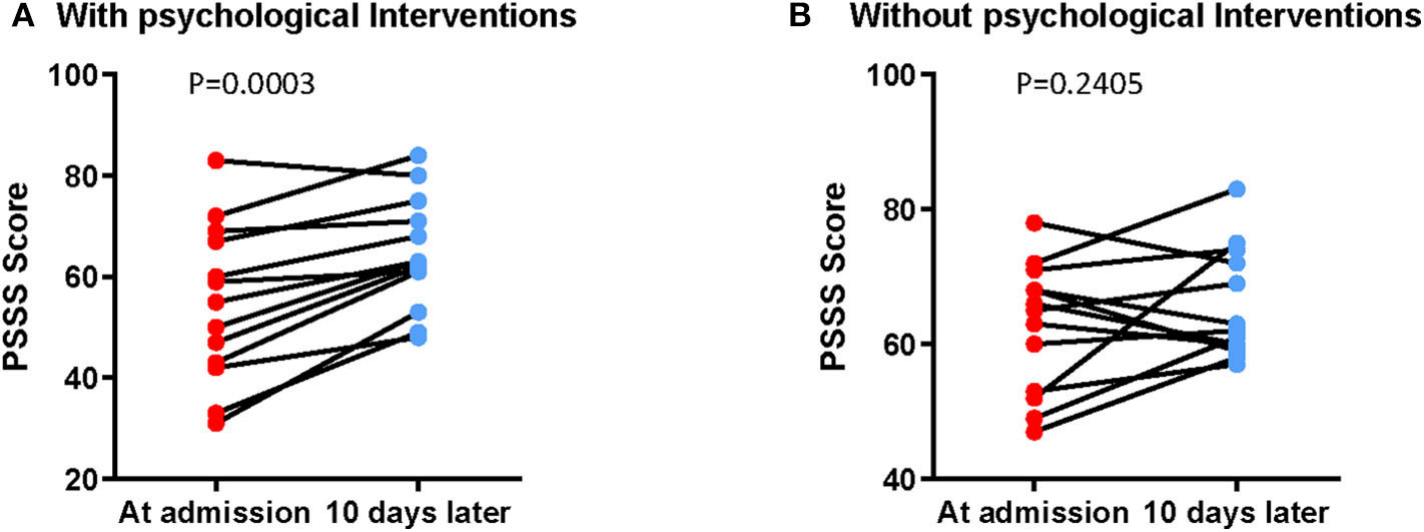
Better self-reported levels of social support by PBI in patients with COVID-19. The alteration of PSSS score in the intervention group **(A)** and control group **(B)**.

We also investigated the level of social support among 26 patients after 10-day treatment. It was found that the PSSS scores were improved after PBI in the intervention group (pre-treatment = 54.69 ± 15.59, post-treatment = 64.46 ± 11.05, *p* < 0.0001), while the PSSS scores in the control group did not alter significantly (pre-treatment = 62.46 ± 9.62, post-treatment = 65.62 ± 8.13, *p* = 0.241) (Figure 3 and Table 7). The results imply that the intervention could enhance patients' perceived social support.

**Table 7:** Comparison of PSSS scores between pre- and post-treatment in the PBI group and the control group.

	**Pre-treatment**	**Post-treatment**	**Mean difference (95% CI)**	***t***	**df**	***p*^a^**
PBI	54.69 ± 15.585	64.46 ± 11.050	−9.769 (−14.065 to −5.474)	−4.955	12	<0.0001
Control	62.46 ± 9.623	65.62 ± 8.130	−3.154 (−8.719 to 2.411)	−1.235	12	0.241

a *Paired Student t-test*.

*PSSS, Perceived Social Support Scale*.

## Discussion

### Prevalence and Factors Linked to Anxiety and Depression in Hospitalized Patients With COVID-19

A number of studies have interlinked depression and anxiety to patients with different diseases (3–5). This study firstly reports the prevalence of anxiety and depression in patients with COVID-19 during the epidemic. The results of the present study showed that 34.72 and 28.47% of patients with COVID-19 had symptoms of anxiety or depression, respectively.

In the present study, it is noteworthy that social support is one of the key factors linked to anxiety and depression for patients with COVID-19 (Table 4). The results show that less social support is correlated with more anxious and depressive symptoms (Table 2). Numerous studies have demonstrated that in the case of disease, patients need more social support, including physical and psychological assistance provided by family members, friends, medical workers, and relevant institutions to cope with difficulty (27). There is consistent evidence that shows that social isolation and loneliness are linked to worse mental health outcomes (28). During the COVID-19 epidemic, many isolated patients often felt helpless and lonely due to the lack of family or friends accompanying them. In such circumstances, medical workers as the major peer support are of great significance to infected patients. In clinical practice, Chinese medical members would keep in touch with patients and try various psychological support methods to help isolated patients rebuild confidence. In some Wuhan makeshift hospitals, patients with mild symptoms did Tai Chi practice [which has been verified as an effective way to improve lung function for COPD patients; (29)] and singing and dancing as physical relaxation, accompanied and guided by medical staff. This kind of doctor–patient interaction may encourage patients to maintain a positive mindset.

Meanwhile, older age and lower oxygen saturation are the other factors considered for patients to be anxious. Previous research has revealed that older patients are at increased risk with severe COVID-19 symptoms and death (26). Additionally, oxygen saturation is a key index to evaluate the severity of patients with COVID-19. According to the Chinese management guideline for COVID-19 (30), patients who have an oxygen saturation ≤93% at rest are defined as severe-type patients. In this study, 11.1% participants were with low oxygen saturation. These results indicate that patients with severe illness are more likely to be anxious. More psychological care and health attention needs to be given to these critically ill patients.

Consistent with previous report, which focused on the psychological responses among general population during the COVID-19 epidemic in China (8), female patients are also prone to developing higher levels of anxiety as shown in the current study. Meanwhile, education background is another associated factor to the mental distress among infected patients. As we expected, family member infection is another factor affecting patients to be depressed. High levels of concern about other family members and lack of family care may magnify pessimism over the illness.

This study shows that hospitalized patients with COVID-19 experience features of anxiety and depression. The significant factors found in the present study may draw medical workers paying more attention to the mental health of patients with COVID-19.

### The Effect of PBI on Patients With COVID-19

Anxiety and depression are related to longer hospitalization (4, 11) and non-adherence to treatment (9, 10) in several diseases. A considerable number of patients with COVID-19 indeed suffered from depression and anxiety, according to the above results. In this study, we conducted a PBI program to investigate its effect on patients with COVID-19.

Due to the fact that COVID-19 is a newly emerging pandemic, few studies on psychological intervention for patients have been reported. In order to make the intervention protocol operable for non-psychological clinical staff, we mainly referred to U.S. SPIKES (17) and Australian Consensus Guidelines (18) on the psychosocial support protocols for disclosing unfavorable information to patients. It is necessary for medical workers to develop relevant communication skills to reduce patients' negative emotions toward their own diseases in clinical practice (30). Meanwhile, it was found that cough (78.5%) and shortness of breath (50.7%) were two of the most common symptoms of COVID-19 in the current study, consistent with other COVID-19 reports (1, 24–26). Breathing exercises have been proven to improve pulmonary function, as well as reduce the levels of anxiety and depression (19, 20). Therefore, we designed the PBI program with psychological support and breathing exercises.

The results showed that anxiety and depression were relieved in the intervention group compared with the control group after PBI, which suggested that PBI effectively reduced anxiety and depression in patients with COVID-19. This might be attributable to the fact that patients in the intervention group received frequent communication with medical staff, which resulted in obtaining more information about the disease and their condition, thereby alleviating the anxiety and fear caused by being blind to the disease. In addition, the self-assessment of social support among 26 patients showed that the PSSS scores were significantly improved after PBI in the intervention group, while the PSSS scores in the control group did not change significantly. The psychological counseling and breathing exercises gave more opportunities for patients to contact other people, which reduced the sense of solitude and let them feel support and concern from others, thereby reducing the psychological distress of patients with COVID-19. This is consistent with the discovery that social support is one of the key factors linked to anxiety and depression for patients with COVID-19. Furthermore, we followed up patients in the intervention group using a discharge questionnaire. All of the 13 patients in the intervention group felt that they received social support and social care a lot, and they experienced the warmth of the society while hospitalized (data not shown).

These findings suggest that PBI, as a way of social support, may have a beneficial effect on COVID-19 patients' mental health. We believe that this program can also be applied to other patients with anxiety and depression. In the setting of non-epidemic, this psychological intervention may have a better effect on patients with sufficient time and diverse methods (such as body language, facial expressions, group discussions, lectures, etc.). Early prevention of mental health problems is of vital importance to help patients have good clinical outcomes and better life quality. As the COVID-19 epidemic continues to spread, our findings are particularly instructive to develop a psychological support strategy for hospitalized patients with COVID-19 in China and other areas affected by the epidemic.

## Study Limitation

It is important to take into account several limitations in this study. For instance, the present study was single-centered; the study sample was not representative of all patients with COVID-19 in China, which limited the generalizability of the results. Due to the restriction of the condition, patients' anxiety and depression assessment was based on a single measurement scale. Additionally, blinding was not feasible for participants and researchers in this study; only the evaluator (who gave the link of questionnaires) and data analyst were blinded for the treatment. Moreover, we found that PBI alleviated anxiety and depression in patients with COVID-19. The PBI program included psychological support and breathing exercises, while the control group only received treatment as usual. Additional evidence is needed to explain whether the effectiveness of PBI is due to the intervention program or more attention offered by medical workers. Lastly, the intervention study has a relatively small number of subjects. A large-scale study is still needed to validate our results.

## Data Availability Statement

The original contributions presented in the study are included in the article/Supplementary Material, further inquiries can be directed to the corresponding author/s.

## Ethics Statement

The studies involving human participants were reviewed and approved by the Research Ethics Commission of Huoshenshan Hospital. Written informed consent to participate in this study was provided by the participants.

## Author Contributions

YD, XK, and TS conceived and designed the study. YC and YL performed the PBI. PJ, KZ, and MT collected the clinical data. FK and JZ analyzed the data. JZ, LD, SW, and XK were involved in drafting the manuscript. YD and TS revised the manuscript. All authors contributed to the article and approved the submitted version.

## Conflict of Interest

JZ, LD, and SW were employed by the company Shanghai Biotecan Pharmaceuticals Co., Ltd. The remaining authors declare that the research was conducted in the absence of any commercial or financial relationships that could be construed as a potential conflict of interest.

## Supplementary Material

Supplementary Figure 1 Photo of COVID-19 patients hospitalized in Huoshenshan Hospital, who were having breathing exercises guided by trained medical workers.
