# Supplementary figures and images for: Effect of Psychological–Behavioral Intervention on the Depression and Anxiety of COVID-19 Patients
Source: Front Psychiatry. 2020 Nov 20;11:586355. doi: 10.3389/fpsyt.2020.586355 (PMC7715028; doi:10.3389/fpsyt.2020.586355)

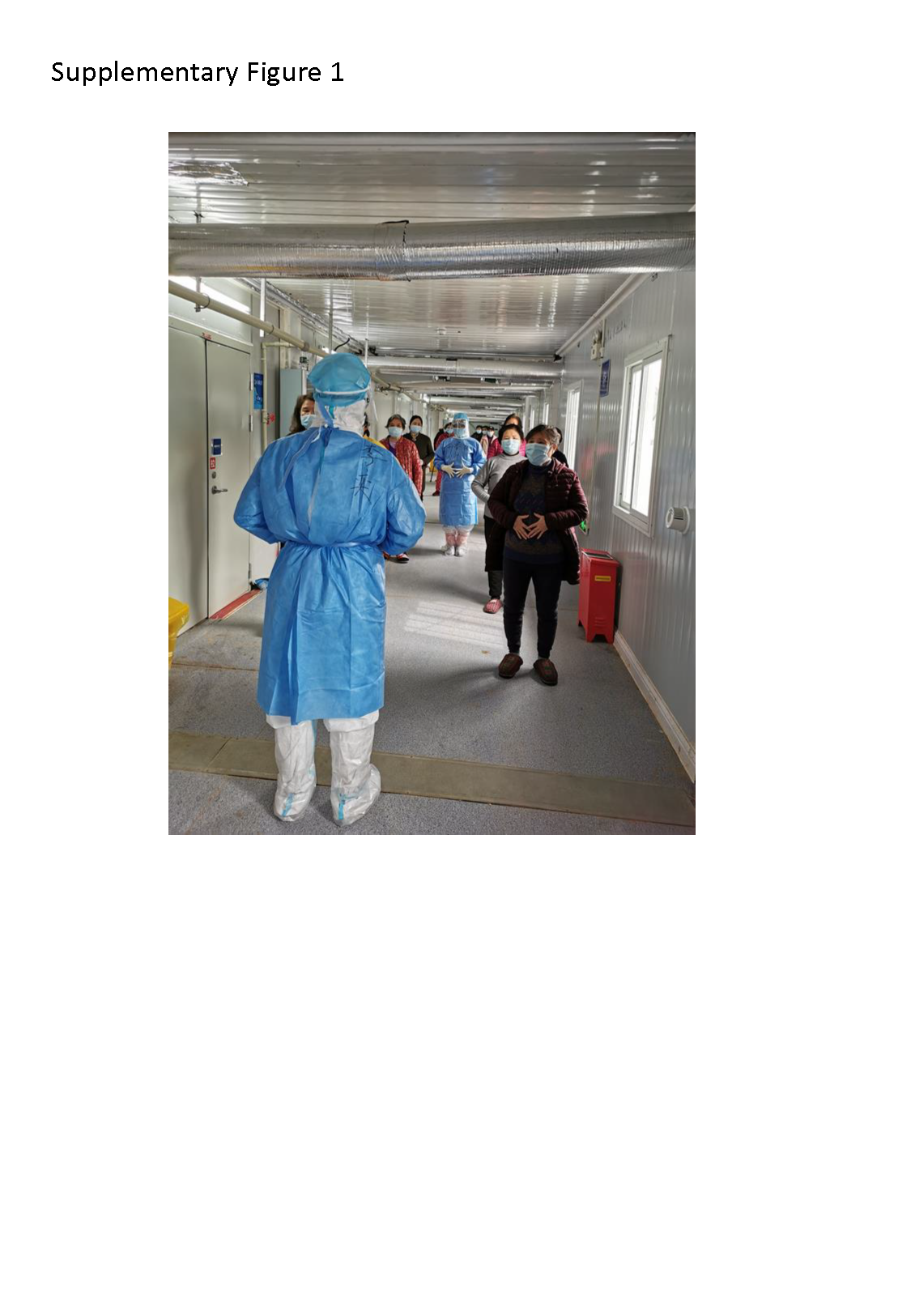

Supplement: Supplementary Figure 1 — Photo of COVID-19 patients hospitalized in Huoshenshan Hospital, who were having breathing exercises guided by trained medical workers. [file Image_1.TIF]
